# Homoepitaxial regrowth habits of ZnO nanowire arrays

**DOI:** 10.1186/1556-276X-6-619

**Published:** 2011-12-07

**Authors:** Jian Liu, Shufan Xie, Yanglong Chen, Xianying Wang, Hongbin Cheng, Fang Liu, Junhe Yang

**Affiliations:** 1 School of Materials Science and Engineering, University of Shanghai for Science and Technology, Shanghai, 200093, China

**Keywords:** ZnO, nanoantenna, photoluminescence, second growth

## Abstract

Synthetic regrowth of ZnO nanowires [NWs] under a similar chemical vapor transport and condensation [CVTC] process can produce abundant ZnO nanostructures which are not possible by a single CVTC step. In this work, we report three different regrowth modes of ZnO NWs: axial growth, radial growth, and both directions. The different growth modes seem to be determined by the properties of initial ZnO NW templates. By varying the growth parameters in the first-step CVTC process, ZnO nanostructures (e.g., nanoantenna) with drastically different morphologies can be obtained with distinct photoluminescence properties. The results have implications in guiding the rational synthesis of various ZnO NW heterostructures.

## Introduction

One-dimensional ZnO nanowires [NWs] have attracted increasing interests for their potential applications in various functional devices, including solar cells [1,2], nanosensors [3], nanogenerators [4], and nano-optoelectronics [5]. Compared with other semiconductor NWs, ZnO has a large band gap (3.37 eV) and high exciton binding energies (60 meV) and thus can be used as ultraviolet laser or light-emitting diodes. In addition, ZnO nanostructures also exhibit the richest morphologies reported so far (e.g., nanocomb, nanoring, nanobridge, nanonail, nanobelt) [2,6-9], making it coveted as building blocks for functional devices. Nonetheless, the growth of ZnO NWs is sensitive to the fabrication conditions and is often difficult to control, i.e., slight changes of the growing parameters may result in drastic differences in morphologies and subsequent semiconducting or optical properties [10]. Although tremendous efforts have been made to understand various growth mechanisms, new morphologies such as NW forests [2], microtowers [11], or micropyramids [12] keep on emerging through regrowth processes, which continue to challenge growth theories and require systematic investigations. Dissimilar to the conventional catalyst-mediated vapor-liquor-solid process, the homoepitaxial growth of ZnO nanostructures on pre-existing ZnO NWs may follow a vapor-solid mechanism since no metal catalysts are involved. It is expected that ZnO NWs with more abundant morphologies can be fabricated using regrowth techniques.

In this work, we investigate the regrowth habits of ZnO NWs using a chemical vapor transport and condensation process [CVTC] [4]. We find that the epitaxial regrowth habits are not only determined by the growth conditions in the second-step CVTC process, but also greatly influenced by the initial morphologies and properties of the ZnO NW templates. When the growth conditions in the first-step CVTC process are changed, the epitaxial regrowth of NWs can either follow axial, radial, or both directions. We demonstrate the successful fabrication of ZnO 'nanoantenna' structures through homoepitaxial regrowth control mechanisms [13]. Our findings offer the new possibility of growing novel nanostructures and have implications in guiding the fabrication of various ZnO NW heterostructures, such as axial p-n junctions and/or core-shell (multi-shell) structures.

## Experimental section

ZnO nanostructures were grown using a two-step CVTC method in a tube furnace (Lindberg blue, Thermo Scientific, Waltham, MA, USA). During the first-step CVTC process, a-plane sapphire substrate was coated with a 1.5-nm-thick Au catalyst. Equal amounts of ZnO powders (99.99%; Alfa Aesar, Ward Hill, MA, USA) and graphite powders (99.9%; Alfa Aesar, Ward Hill, MA, USA) were loaded into an alumina boat to provide Zn vapors. Argon flow with a rate of 12 sccm was used as the carrier gas. The growth temperature was set at 910°C with a ramping rate of 50°C/min. The initial growth times of samples A, B, and C were 10 min, 10 min, and 30 min, respectively. Before growth, the Au catalyst film of sample B was exposed to a focused ion beam [FIB] with a Ga+ ion source (30 KeV, 0.19 nA, 900 ms). After the first CVTC process, ZnO NWs were taken out and used as the templates for the second-time CVTC growth. The second-time growth parameters are nearly identical to those in the first step, except that the growth time of all the samples was set to 10 min. The Third- and fourth-time growths of sample A follow the same growth conditions as those used in the second-time growth.

The morphologies of all samples were characterized by scanning electron microscopy [SEM] (FEI Quanta FEG, FEI Co., Hillsboro, OR, USA). Room temperature photoluminescence [PL] spectra were collected using Flurolog-3-p type spectrometer in the air. The crystal structures and orientations were analyzed using X-ray diffractometry [XRD] (D8 ADVANCE, Bruker AXS GmbH, Karlsruhe, Germany) and transmission electron microscopy [TEM] (FEI Tecnai G2, FEI Co., Hillsboro, OR, USA).

## Results and discussions

**Figure 1:**
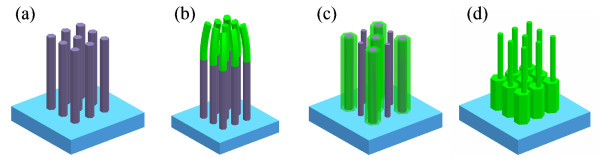
**Schematic diagrams of possible regrowth modes**. (**a**) ZnO NW templates after the first-time CVTC; (**b**) axial growth mode; (**c**) radial growth mode; (**d**) both axial and radial growth mode; (b, c, d) represent three different epitaxial growth modes during the regrowth. As a result, nanoantennas are formed.

Through extensive growth and regrowth experiments, we observed three different types of epitaxial regrowth modes, which are schematically illustrated in Figure 1. Figure 1a shows the ZnO NW templates after the first-step CVTC. Figure 1b, c, d represents ZnO NW arrays of samples A, B, and C after the second-time CVTC growth, respectively. The regrowth of sample A is dominated by growth along the axial direction. In contrast, the epitaxial regrowth of sample B tends to be along the radial direction. For sample A, as the NWs grow longer, they start to incline and bundle together at the very top. Finally, for sample C, NWs grow along both radial and axial directions; moreover, reconstructions or self-assembling of the ZnO NW templates seem to have occurred during the second-time growth.

**Figure 2:**
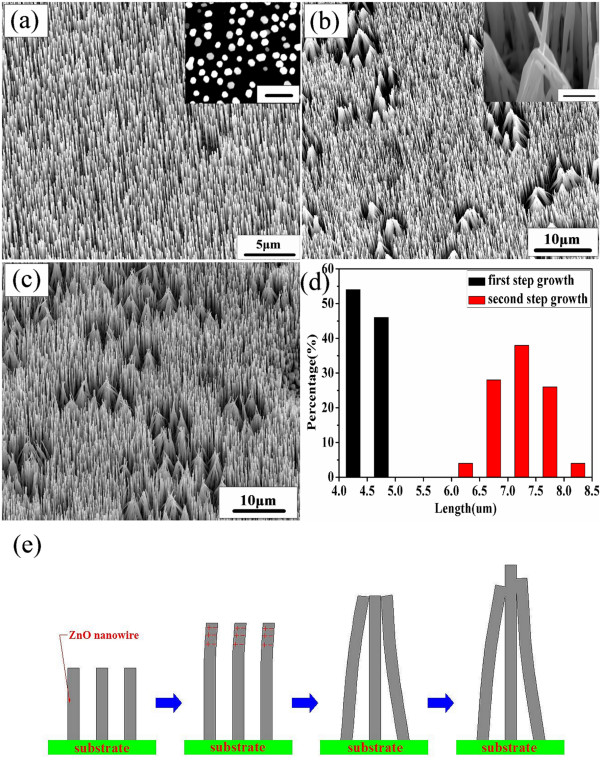
**Sample A**. SEM images of ZnO NW arrays after the (**a**) first-, (**b**) second-, and (**c**) fourth-time growths. The growth time is 10 min for each cycle. NW bundling is observed in both (b) and (c). A zoomed-in image of the bundle is shown in the inset of (b). The scale bar is 1 μm. (**d**) Statistic diameter distribution of ZnO NWs after the first- and second-time growths. (**e**) A possible mechanism that leads to bundling process of ZnO NWs during the second-time growth.

To experimentally illustrate the three different regrowth modes above, Figure 2 shows the 35° tilt-view SEM image of sample A after the first-time growth (Figure 2a), second-time growth (Figure 2b), and fourth-time growth (Figure 2c). In Figure 2a, the as-deposited ZnO NWs are vertically aligned with the diameters around 100 to 120 nm and lengths up to 4 to 5 μm. After the second-time growth, the NWs become longer, and most of them remain aligned with some visibly bundled NW tops (Figure 2b). Interestingly, the NW diameters are hardly changed with the lengths extended to approximately 7 to 8 μm, as can be seen from the length distribution histograms in Figure 2d. The inset in Figure 2b gives a zoomed-in view of the bundled tips, indicating that the bundling occurs during the growth after which some NW growth terminates. The repeated growth of sample A under the same growing conditions for the third and fourth time shows similar results. After each circle, the NWs become longer, and more bundles appear (Figure 2c). The inset of Figure 2a illustrates the hexagonal shapes of the top surfaces, suggesting that Au nanoparticles are completely consumed after the 10-min growth. Therefore, the homoepitaxial growth along the axial direction was not caused by the Au catalyst. Under a thermodynamic equilibrium state, ZnO NWs tend to be epitaxial with the existing NWs in order to retain the lowest system energy. Thus, the regrowth behavior of sample A is understandable.

Bending, bundling, or coiling of ZnO NW arrays are commonly reported in the literature. Electrostatic interactions due to the polar <0001> surface with different termination atoms were considered as the main cause of bending and coiling [6]. This mechanism, however, may have difficulties in understanding why most NW bundles observed here are touched from side surfaces (i.e., ±(1-210) surface). We speculate, in Figure 2e, that while the NWs grow longer, some of them tend to incline due to the geometric instability. Since ZnO NWs have piezoelectric properties, this causes the side surfaces to be either positively or negatively charged. The opposite charges exist depending on the stretching or compression of the NWs, which could lead to the attraction of NW tops because of the large electrostatic force.

**Figure 3:**
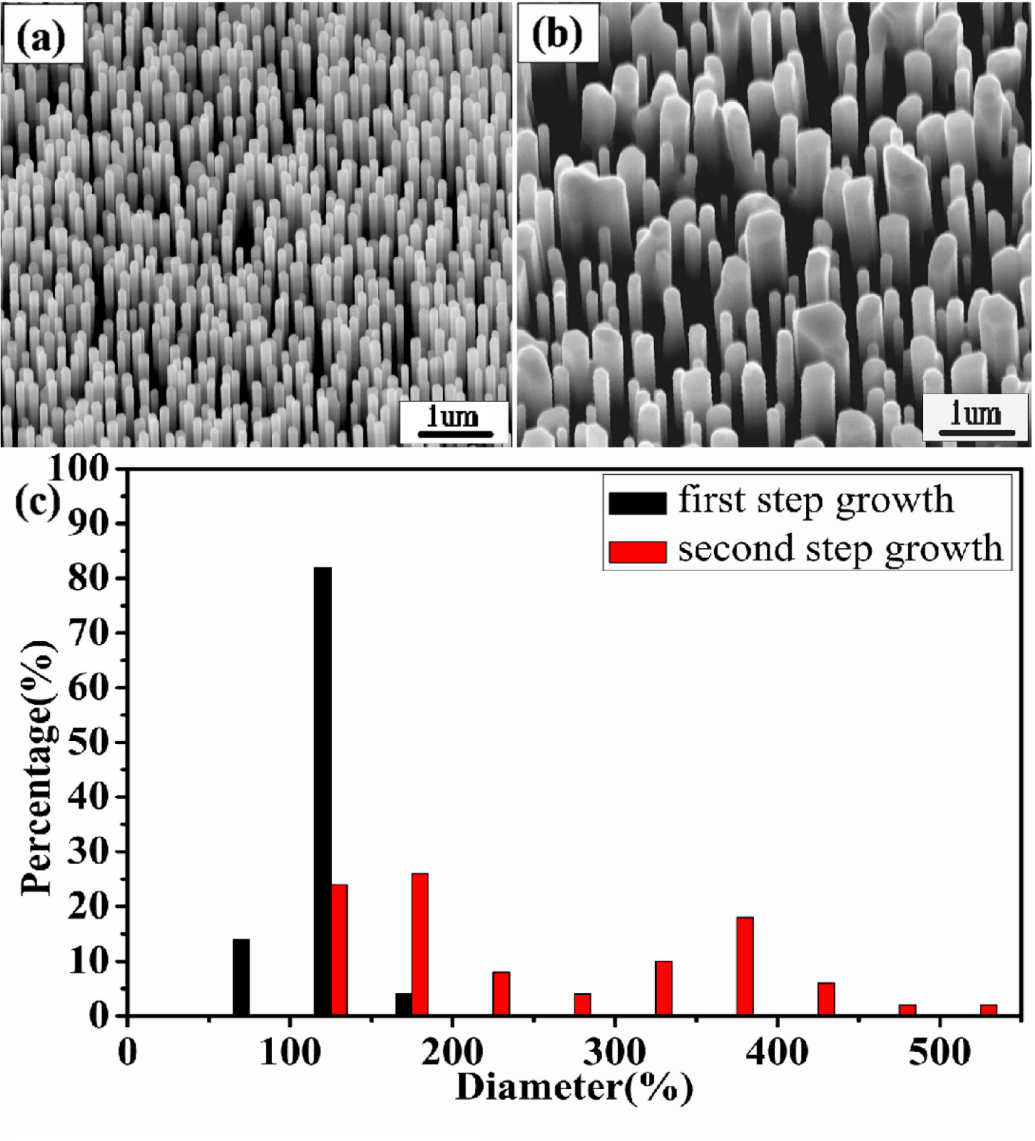
**Sample B**. SEM images of ZnO NWs after the (**a**) first- and (**b**) second-time growths and (**c**) their corresponding diameter distributions, respectively.

Interestingly, regrowth habit along the radial direction was also observed in our experiments. Figure 3 shows the regrowth behavior of sample B, whose growth conditions were the same with those of sample A, except that the Au film was pre-exposed to FIB. As seen from the SEM image in Figure 3a, after the first-step CVTC process, uniform ZnO NWs with an average diameter of 110 nm are synthesized. After regrowth, however, the morphologies of the NWs have changed drastically. Although still vertically aligned, the NWs show abnormal growth along radial directions. The diameters are measured in the range of approximately 107 to 504 nm with the lengths virtually unchanged, as shown in Figure 3b, c. These results unambiguously demonstrate that the preferential growth direction of the NWs is along the radial direction. Furthermore, we observed that the rectangular shape of the NWs, possibly due to the cold-welding of several NWs, is consistent with the radial growth mode observed. Interestingly, the diameters of sample B increased to larger than 1 μm and became more widely distributed after cyclic regrowth for the third and fourth times, again suggesting that radial epitxial regrowth is preferred.

The detailed mechanism why ZnO nanowires have preferential regrowth along the radial direction remains unclear. Ga^+ ^ion implantation in the Au film during the FIB processing might be one of the important factors. Recent experiments in the hydrothermal growth of ZnO NWs suggest that positively charged Ga generally tends to segregate along the side surface of the ZnO NWs [14], which may catalyze the radial growth of the ZnO NWs since Ga-Zn metal has a very low eutectic point.

**Figure 4:**
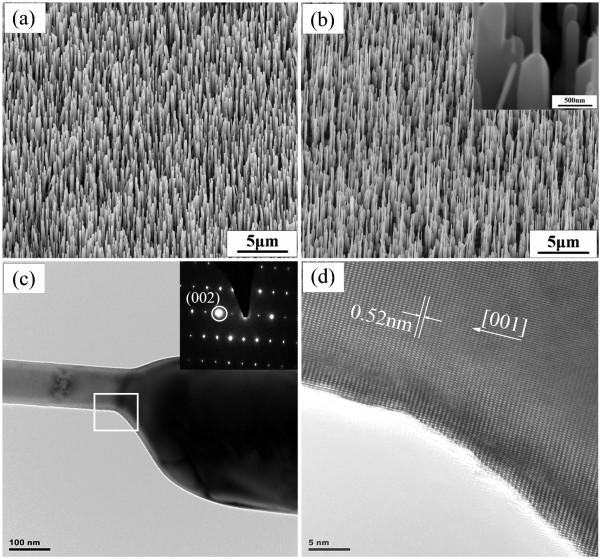
**Sample C**. SEM images of ZnO NWs after the (**a**) first- and (**b**) second-time growths. Nanoantennas are observed. The inset in (b) is a zoomed-in image of a nanoantenna. (**c**) Bright-field TEM image of a nanoantenna and SAED patterns (inset) taken from tip, base, and junction areas. (**d**) High-resolution TEM image of the red-rectangular area in (c).

Besides the axial and radial epitaxial growth, we observed the third regrowth behavior. Antenna-like NWs were obtained through the regrowth of 30-min deposited NWs. As shown in Figure 4a, the initial NWs have an average length of approximately 4 to 5 μm and an average diameter of approximately 150 nm. We note that the average length of this sample after a 30-min growth is similar to that of samples grown at 10 min; however, the diameter distributions are much broader. We speculate that, after such a long time growth, the ZnO powder source may have been exhausted, leading to an additional annealing process that could affect the NW diameter distributions. Figure 4b exhibits the SEM image of the NWs after regrowth under the same second-step growth conditions. Interestingly, most NWs become nanoantenna shapes which can be better discerned in the inset of Figure 4b. The definition of nanoantenna comes from its geometrical resemblance to the antenna of a walkie-talkie or an old style cell phone. These nanoantennas are seen for the first time in ZnO. Nanostructures such as nanoantennas or nano-obelisks are considered as one of the best geometries for gas-sensing or field-emission applications [13,15]. From Figure 4b, the length of the nanoantenna base is approximately 1.5 μm, and the diameter ranges from 300 nm to 900 nm. The diameters of the tips are substantially smaller, in the range of approximately 50 to 100 nm. The TEM image shown in Figure 4c indicates that the nanoantenna has an asymmetric geometry, with the tip sitting near one side of the base. Selected area electron diffraction [SAED] taken from the tip, base, and junction areas shows the same single-crystalline patterns (inset of Figure 4c). The high-resolution TEM image in Figure 4d confirms the single crystalline structure with a 0.52-nm lattice spacing (i.e., *c*-axis growth direction).

We notice that the base lengths of nanoantennas are shorter than those of the original NW templates, suggesting that in addition to simultaneously axial and radial growth, diffusion-assisted NW self-assembling has occurred. Our static annealing experiments of NWs grown at 30 min have indeed revealed the shortening and widening of the NWs. This offers a plausible explanation of the shorter bases we see in nanoantennas. During the forming process of the bases, the tops of relatively longer NWs remain unmelted and protrude out of the bases, which act as the nucleation centers and promote the growth of nanoantenna tips. The asymmetric positioning of the tips relevant to their bases suggests that the mechanism of the nanoantenna is likely to be distinguished from the formation mechanism of microtowers reported in the literature [11].

**Figure 5:**
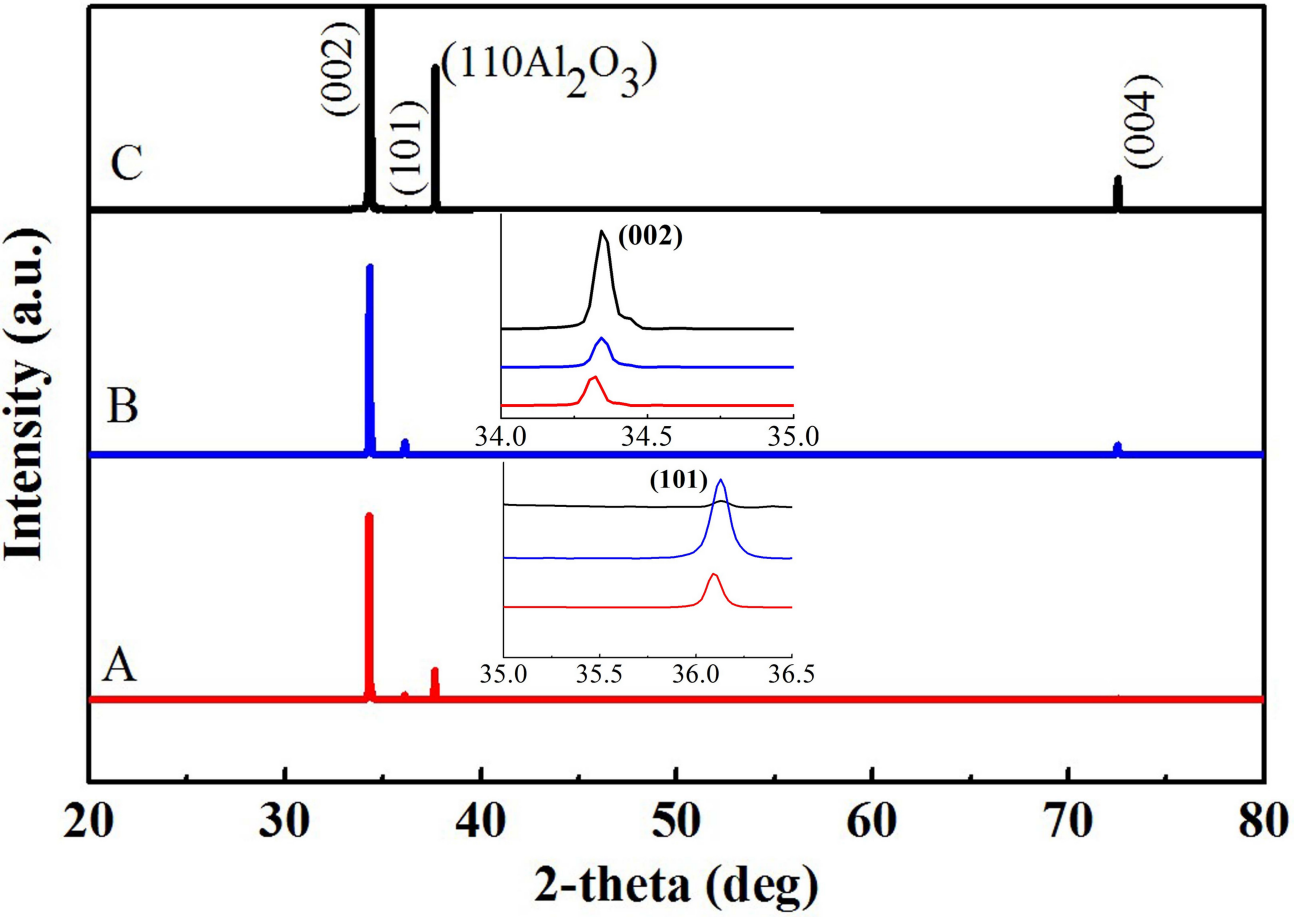
**X-ray diffraction patterns of samples A, B, and C after regrowth**.

To further decode the growth and/or regrowth mechanisms of the above three modes, we used XRD to investigate the crystal structures of all samples after first- and second-time growths. After the second-time growth and as shown in Figure 5, all three samples have strong (002) peaks, indicative of dominant basal plane growth mechanism. In addition, the samples also show a weak (101) peak, suggestive of the secondary growth direction of the NWs. To distinguish the subtle differences, we calculate the intensity ratios of the (101)/(002) peaks for samples A, B, and C, which show values of 13.0%, 2.3%, and 4.7% after the first-time growth, and 4.5%, 7.2%, and 0.3% after regrowth, respectively. The appreciable increase of the (101)/(002) intensity ratio in sample B suggests that the radial growth mode favors the (101)-oriented NWs. In contrast, both sample A and nanoantennas (i.e., sample C) clearly prefer the (002) orientation, as indicated from the reduced intensity ratios. The results imply that axial growth mode favors the basal plane. These interesting observations could help us to selectively grow nanoantennas for field-emission or gas-sensing type of applications.

**Figure 6:**
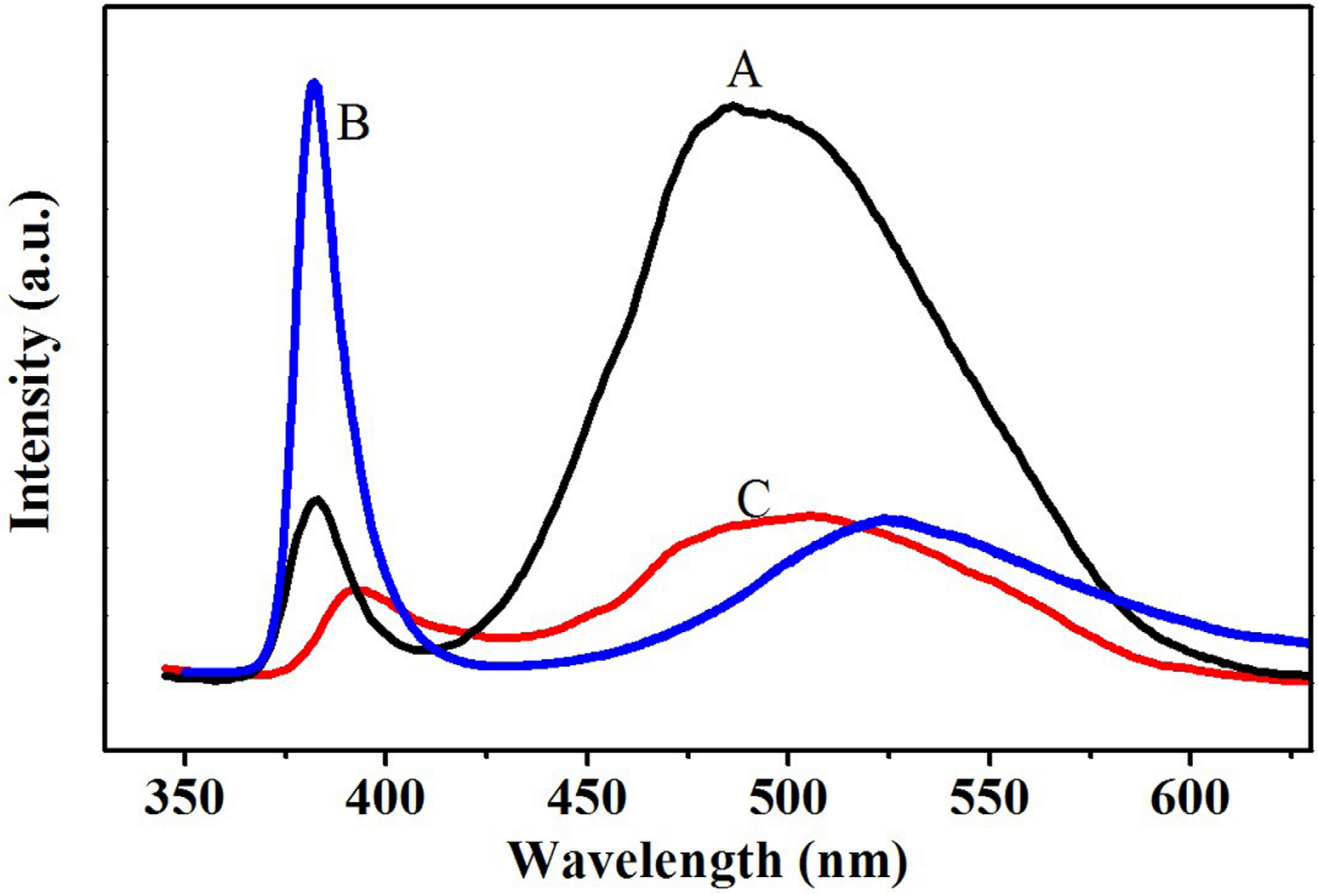
**Room temperature PL spectra of samples A, B, and C**.

To further examine the crystal quality and PL properties, Figure 6 shows the room temperature PL spectra of the three samples. The excitation laser wavelength is 325 nm. It is well known in the literature that CVTC-grown ZnO NWs typically have a band-edge emission peak centered at approximately 380 nm and a green emission peak at approximately 520 nm. The green emission peak is also referred to as the deep-level or trap-state emission that is associated with a defect state (such as oxygen vacancies) in ZnO NWs. The relative intensity ratio of these two peaks often has implications on the crystal quality and trapped defect conditions. On that note, sample B has a relatively weak green emission peak, whereas both samples A and C have a very strong defect emission peak, likely due to trapped surface or subsurface oxygen vacancies. We contribute this effect to the higher surface-volume ratios of thinner NWs in sample A and nanoantennas (i.e., sample C). These observations are consistent with those reported in the literature [10]. Our results have suggested strongly that different regrowth modes can be taken advantage of in order to tune the optical properties of ZnO NWs/nanoantennas for optoelectronics or sensing applications.

## Conclusions

We demonstrate that the regrowth of ZnO NWs by a two-step CVTC process can lead to NWs with different morphologies and properties. By varying the growth parameters in the first-step CVTC process, ZnO NWs can preferentially grow along the axial, radial or both directions. Unlike Si or Ge NWs where axial or core-shell heterostructures can be fabricated by varying different gaseous precursors, most ZnO NWs are grown by evaporating solid ZnO powder which makes it difficult to switch the precursor. Our two-step CVTC processes suggest a simple yet effective method to fabricate axial or core-shell ZnO NW heterostructrues, such as p/n ZnO junctions. Moreover, ZnO nanoantennas first reported here can be readily obtained by our two-step CVTC process and are good candidates for field emitters and/or nanosensors.

## Competing interests

The authors declare that they have no competing interests.

## Authors' contributions

JL and XYW conceived the study. JL, SFX, and YLC carried out the experiments. XYW drafted the manuscript. All authors are involved in revising the manuscript and approved the final version.
